# Hydrocephalus caused by conditional ablation of the *Pten *or *beta-catenin *gene

**DOI:** 10.1186/1743-8454-5-16

**Published:** 2008-10-18

**Authors:** Akihira Ohtoshi

**Affiliations:** 1 Center for Molecular Neurobiology, The Ohio State University, 1060 Carmack Road, Columbus, OH 43210, USA

## Abstract

To investigate the roles of Pten and β-Catenin in the midbrain, either the *Pten *gene or the *β-catenin *gene was conditionally ablated, using *Dmbx1 *(diencephalon/mesencephalon-expressed brain homeobox gene 1)*-Cre *mice. Homozygous disruption of the *Pten *or *β-catenin *gene in *Dmbx1*-expressing cells caused severe hydrocephalus and mortality during the postnatal period. Conditional deletion of *Pten *resulted in enlargement of midbrain structures. *β-catenin *conditional mutant mice showed malformation of the superior and inferior colliculi and stenosis of the midbrain aqueduct. These results demonstrate that both Pten and β-Catenin are essential for proper midbrain development, and provide the direct evidence that mutations of both *Pten *and *β-catenin *lead to hydrocephalus.

## Findings

Congenital hydrocephalus is one of the most common birth defects associated with malformation and/or malfunction of the brain. Although genetic factors are likely to be involved in pathogenesis of hydrocephalus, molecular etiology that causes congenital hydrocephalus is poorly understood, partly due to involvement of multiple genes [1]. Recent studies implicate the genes *Pten *and *β-catenin *in association with hydrocephalus [2-4]; however, it is not clear whether mutations of these genes are causally involved in hydrocephalus. This report shows that conditional inactivation of either *Pten *or *β-catenin *causes hydrocephalus in mice. Although the relationship between *Pten *and *β-catenin *has been intensively investigated in cancer cells in relation to tumorigenesis, it is not known how these genes interact, in terms of brain development.

Pten is a phosphatase that plays critical roles in intracellular signal transduction through dephosphorylation of substrates such as Akt and S6 kinases [5]. Although *Pten *is well known as a tumor suppressor gene, it is also involved in normal cellular proliferation/differentiation and function. Conditional ablation of the *Pten *gene by *Nestin-Cre *mice revealed that Pten is important for proper neural stem cell proliferation and maintenance of soma size [6]. Ablation of *Pten *by *Gfap-Cre *mice causes neuronal hypertrophy and behavioral abnormalities similar to Lhermitte-Duclos disease [7,8].

β-Catenin acts in both cadherin-catenin cell adhesion and Wnt signalling pathways and plays a crucial role in multiple physiological processes such as embryogenesis and cancer. Deletion of *β-catenin *in *Wnt1*-expressing cells demonstrated its essential function in embryonic brain development [9]. Inactivation of *β-catenin *by *Nestin-Cre *mice revealed that β-Catenin is also required for morphogenesis of the cerebellum [10].

**Figure 1:**
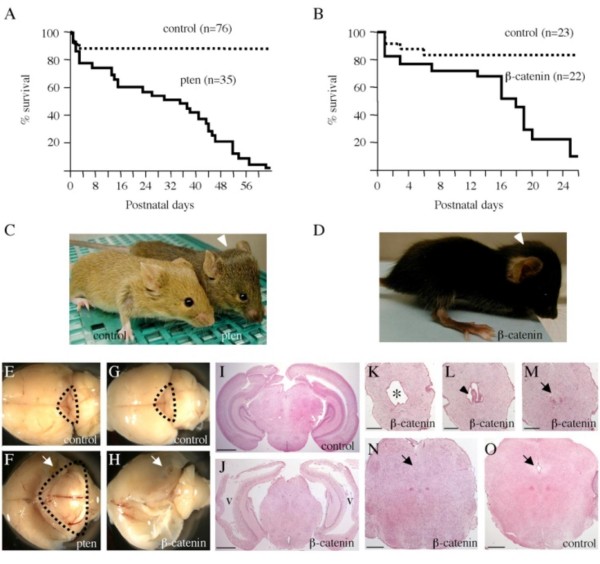
**Mortality and hydrocephalus of *Pten *and *beta-catenin *conditional mutant mice**. A, B: Kaplan-Meier survival curves of *Pten*^*loxP*/*loxP*^; *Dmbx1-Cre*, *β-catenin*^*loxP*/*loxP*^; *Dmbx1-Cre *and their littermate control mice. C, D: Overt appearance of pten and β-catenin mutant mice. Note that mutant mice manifest an enlarged head (white arrowheads). E-H: Dorsal view of the brains dissected from pten, β-catenin mutant and their littermate control mice. Cerebral cortex of the mutant mice was paper-thin due to the ventricular dilatation of hydrocephalus (white arrows). Dotted areas indicate superior and inferior colliculi. The β-catenin mutant mice lack these structures. E and F, postnatal day (P) 37; G and H, P 24. I, J: Coronal sections of β-catenin control and mutant brains at P 7. The β-catenin mutant brain has dilated lateral (v) and third ventricles and a thinner cortex. Scale bars, 1 mm. K-O: Coronal sections of the midbrain from β-catenin mutant and control mice at P 7. In the rostral sections of the β-catenin mutant brain, dilatation (asterisk) and abnormal protrusion (black arrowhead) in the midbrain aqueduct were observed. In the caudal sections, normal midbrain aqueduct was not detected in the mutant mice (black arrows). Scale bars, 500 μm.

To investigate phenotypes manifested by disruption of the *Pten *or *β-catenin *gene in the midbrain, *Pten*^*loxP*/*loxP *^and *β-catenin*^*loxP*/*loxP *^mice were obtained from the Jackson laboratory [6,9] and crossed with *Dmbx1-Cre *mice that express Cre recombinases in the mesencephalon/midbrain regions of the developing nervous system [11]. First, *Pten*^*loxP*/*loxP *^and *β-catenin*^*loxP*/*loxP *^mice were mated with *Dmbx1-Cre *mice to produce *Pten*^*loxP*/*wt*^*; Dmbx1-Cre *and *β-catenin*^*loxP*/*wt*^*; Dmbx1-Cre *mice. Then, these mice were intercrossed to generate *Pten*^*loxP*/*loxP*^*; Dmbx1-Cre *and *β-catenin*^*loxP*/*loxP*^*; Dmbx1-Cre *mice. The institutional animal care and use committee approved the animal studies. Heterozygous deletion of the *Pten *or *β-catenin *gene in *Dmbx1*-expressing cells did not develop any overt phenotype; however, all homozygous deletion mice died during the early postnatal period with progressive enlargement of the head (Figs. 1A–D). Median survival times of *Pten *and *β-catenin *mutant mice were 23 and 16 days, respectively. The length of survival ranged from 1 day to 63 days (*Pten*) and 1 day to 28 days (*β-catenin*). All surviving pups displayed the apparent abnormal head around 10 days after birth, mobility impairment and poor growth that are typical phenotypes caused by hydrocephalus. Anatomical examination of the brains confirmed hydrocephalus, dilatation of lateral ventricles and a remarkably thinned cerebral cortex (Figs. 1E–J). The *Pten*^*loxP*/*loxP*^*; Dmbx1-Cre *mice had the massive midbrain, which is a consistent phenotype with hypertrophic brains seen in *Pten*-deficient mice [6-8]. The midbrain contains a narrow canal communicating between the third and fourth ventricles and its stenosis often leads to obstruction of cerebrospinal fluid (CSF) flow. Continuous expansion of soma size of *Dmbx1*-expressing cells presumably causes non-communicating hydrocephalus in the *Pten*^*loxP*/*loxP*^*; Dmbx1-Cre *mice. The *β-catenin*^*loxP*/*loxP*^*; Dmbx1-Cre *brain lacked normal midbrain structures including superior and inferior colliculi. Histological analyses revealed the presence of a protruding structure of the ependymal wall and the disappearance of the midbrain aqueduct (Figs. 1K–O). The malformation of the midbrain aqueduct probably causes obstructive hydrocephalus in the *β-catenin*^*loxP*/*loxP*^*; Dmbx1-Cre *mice.

These results suggest that Pten and β-Catenin are required for brain formation and their loss of function results in aberrant brain development, progressive hydrocephalus and the postnatal lethality. Recently, a mutation of the *PTEN *gene is implicated in association with human VATER-hydrocephalus syndrome [2]. In *hyh *(hydrocephalus with hop gait) mutant mice, abnormal localization of cell fate determinant proteins such as β-Catenin and E-cadherin was observed in neuroepithelial cells [3]. Mislocalization of β-Catenin and N-cadherin was also observed in *Dlg5 *mutant mice that manifest obstructive hydrocephalus [4]. These observations suggest that Pten and β-Catenin are associated with congenital hydrocephalus. Here, direct evidence demonstrates that loss of Pten or β-Catenin causes hydrocephalus in mice.

Although phenotypical manifestations such as dilated ventricles, excessive CSF and mortality are commonly observed in animal models with both communicating and non-communicating hydrocephalus, the molecular and cellular etiologies are diverse. The *hyh *mutant mice carry a mutation in the *α-SNAP *gene that encodes a protein involved in SNAP receptor (SNARE)-mediated apical membrane transport of cadherin/catenin complexes in polarized epithelial cells [3]. Dlg5 is also required for SNARE-dependent intracellular trafficking of cadherin/catenin molecules and disruption of *Dlg5 *results in collapse of epithelial tubes [4]. Therefore, loss of β-Catenin in epithelial cells likely caused the stenosis of the midbrain aqueduct in the *β-catenin*^*loxP*/*loxP*^*; Dmbx1-Cre *mice. Alternatively, the aqueduct closure could be secondary to disturbance of CSF flow as seen in Hydrocephalus Texas (H-Tx) rats that show abnormalities in secretory ependymal cells of the subcommissural organ [12]. Severely affected H-Tx rats die at 4–6 weeks whereas the *β-catenin*^*loxP*/*loxP*^*; Dmbx1-Cre *mice did not survive beyond 4 weeks. The *hy3 *mice carry a mutation in the *Hydin *gene that is expressed in the ciliated ependymal cells and die before 7 weeks of age [13]. They first develop a defect in CSF reabsorption and later a blockage within the cerebral aqueduct. Further investigations are needed to elucidate the pathogenic mechanisms leading to hydrocephalus in the *Pten*^*loxP*/*loxP*^*; Dmbx1-Cre *and *β-catenin*^*loxP*/*loxP*^*; Dmbx1-Cre *mice. These mutant mice will serve as a novel model for congenital hydrocephalus and provide a novel opportunity to investigate molecular etiology of hydrocephalus.

## Competing interests

The author declares that AO has no competing interests.

## Authors' contributions

AO designed and carried out experiments, and prepared the manuscript. The author has read and approved the final version of the manuscript.

## References

[B1] Zhang J, Williams MA, Rigamonti D (2006). Genetics of human hydrocephalus. J Neurol.

[B2] Reardon W, Zhou XP, Eng C (2001). A novel germline mutation of the PTEN gene in a patient with macrocephaly, ventricular dilatation, and features of VATER association. J Med Genet.

[B3] Chae TH, Kim S, Marz KE, Hanson PI, Walsh CA (2004). The hyh mutation uncovers roles of αSnap in apical protein localization and control of neural cell fate. Nat Genet.

[B4] Nechiporuk T, Fernandez TE, Vasioukhin V (2007). Failure of epithelial tube maintenance causes hydrocephalus and renal cysts in Dlg5^-/- ^mice. Dev Cell.

[B5] Manning BD (2004). Balancing Akt with S6K: implications for both metabolic diseases and tumorigenesis. J Cell Biol.

[B6] Groszer M, Erickson R, Scripture-Adams DD, Lesche R, Trumpp A, Zack JA, Kornblum HI, Liu X, Wu H (2001). Negative regulation of neural stem/progenitor cell proliferation by the Pten tumor suppressor gene in vivo. Science.

[B7] Kwon CH, Zhu X, Zhang J, Knoop LL, Tharp R, Smeyne RJ, Eberhart CG, Burger PC, Baker SJ (2001). Pten regulates neuronal soma size: a mouse model of Lhermitte-Duclos disease. Nat Genet.

[B8] Backman SA, Stambolic V, Suzuki A, Haight J, Elia A, Pretorius J, Tsao MS, Shannon P, Bolon B, Ivy GO, Mak TW (2001). Deletion of Pten in mouse brain causes seizures, ataxia and defects in soma size resembling Lhermitte-Duclos disease. Nat Genet.

[B9] Brault V, Moore R, Kutsch S, Ishibashi M, Rowitch DH, McMahon AP, Sommer L, Boussadia O, Kemler R (2001). Inactivation of the β-catenin gene by Wnt1-Cre-mediated deletion results in dramatic brain malformation and failure of craniofacial development. Development.

[B10] Schüller U, Rowitch DH (2007). β-catenin function is required for cerebellar morphogenesis. Brain Res.

[B11] Ohtoshi A, Bradley A, Behringer RR, Nishijima I (2006). Generation and maintenance of Dmbx1 gene-targeted mutant alleles. Mamm Genome.

[B12] Somera KC, Jones HC (2004). Reduced subcommissural organ glycoprotein immunoreactivity precedes aqueduct closure and ventricular dilatation in H-Tx rat hydrocephalus. Cell Tissue Res.

[B13] Davy BE, Robinson ML (2003). Congenital hydrocephalus in hy3 mice is caused by a frameshift mutation in Hydin, a large novel gene. Hum Mol Genet.

